# Outcomes During the Learning Curve and Feasibility of Implementing the European Hernia Society Recommendation Guidelines for Robotic Abdominal Wall Surgery Within a UK Centre

**DOI:** 10.3389/jaws.2025.15008

**Published:** 2025-08-06

**Authors:** Javed Latif, Matthew Brazkiewicz, Ihsan Inan, Filip Muysoms, Imran Bhatti, Altaf Awan

**Affiliations:** ^1^ Department of Robotic Pancreaticobiliary and Abdominal Wall Reconstruction Unit, University Hospitals of Derby and Burton, Derby, United Kingdom; ^2^ Department of General and Visceral Surgery, Clinique Generale Beaulieu, Geneve, Switzerland; ^3^ Department of Surgery, Maria Middalares, Gent, Belgium

**Keywords:** European Hernia Society, hernia repair, robotic-assisted surgery, robotic training program, robotic abdominal wall surgery

## Abstract

**Background:**

Robotic-assisted surgery (RAS) for abdominal wall hernia repair is an established, minimally invasive technique that is in the early phase of adoption within the UK. We aimed to demonstrate the impact on patient outcomes and safety of hernia repair by adhering to the robotic abdominal wall surgery pathway developed by the European Hernia Society.

**Materials and Methods:**

Two experienced laparoscopic surgeons in the UK underwent four phases that involved preclinical and clinical phases. The surgeons performed RAS hernia surgery with a stepwise increase in complexity, from robotic transabdominal preperitoneal (rTAPP) inguinal hernia repair, robotic transabdominal retrorectus umbilical prosthesis (rTARUP)/extended totally extraperitoneal (eTEP) to robotic transversus abdominis release (rTAR).

**Results:**

In total, 144 patients underwent RAS for hernia repair. Of these, 97 underwent rTAPP inguinal hernia repair (23 bilateral cases). The median operative time was 56 min for unilateral and 101 min for bilateral repair. Four (3.3%) rTAPP patients experienced complications, with two recurrences at the 6-month follow-up. Forty-two patients underwent rTARUP/eTEP repair, with a median operative time of 167 min. Two (4.8%) of these patients experienced postoperative complications. No recurrences were observed at 6 months. Thirteen patients with incisional hernias underwent rTAR, with a median operative time of 426 min. No recurrences were observed in rTAR patients, followed for up to 12 months.

**Discussion:**

Implementation of the EHS training pathway for robotic abdominal wall surgery resulted in a low complication rate and satisfactory clinical outcomes and represents a robust mechanism for surgeons to safely adopt complex robotic abdominal wall surgery.

## Introduction

Robotic-assisted surgery (RAS) is changing the landscape of general surgery by encouraging widespread adoption of minimally invasive surgery (MIS), especially in the discipline of hernia repairs [1]. Traditionally, MIS for ventral hernias has involved the closure of the defect and placement of an intraperitoneal onlay mesh (IPOM), known as the IPOM plus repair [2]. However, despite the use of a coated mesh there is always a risk of bowel adherence, adding to complexity of future abdominal surgery [3, 4]. Furthermore, fixation of the mesh with tacks can cause significant pain in the immediate postoperative period and at follow-up [5]. RAS enables dissection in the retromuscular Rives-Stoppa plane (transabdominal retrorectus umbilical prosthesis (rTARUP) or extended, totally extraperitoneal (eTEP) in defects ≤5 cm) and beyond the lateral border of the rectus sheath, also known as EIT ambivium (transabdominal or eTEP transversus abdominis release (TAR) for defects >5 cm) [6]. This allows tension-free closure of the hernia defect(s) and placement of a large mesh in the extraperitoneal plane avoiding contact with intra-abdominal viscera.

The benefit of performing RAS ventral hernia repair has been established and reported to be more substantial with increasing complexity of repair [7]. Some of the advantages include reduced length of stay and a decreased risk of recurrence and wound complications [8, 9]. These techniques are difficult to emulate with conventional laparoscopy and ergonomically can be challenging [10, 11]. However, they have been performed by a few specialist hernia surgeons, who have reported safe and efficacious outcomes [11, 12].

Accessibility of RAS for surgeons naïve to complex, ventral hernia MIS (rTARUP and rTAR) necessitates a structured training pathway to achieve both safety and proficiency. The European Hernia Society (EHS) has developed recommendations describing the curriculum of the training pathway for robotic abdominal wall surgery (RAWS), with the aim of safeguarding patients from harm while surgeons progress through the learning curve [7]. The guidance aims to play an important role in the clinical phase of training (phases 3 and 4) by promoting a stepwise progression in complexity, from robotic (r)TAPP inguinal hernia repair to rTARUP/eTEP and finally to rTAR and eTEP-TAR. Progression is bolstered by courses where the procedures are performed using either wet or live animal models, placing specific attention on relevant surgical anatomy and execution of procedural steps for complex abdominal wall reconstruction. Initial cases of a procedure are proctored by an expert robotic hernia surgeon, to ensure that the skills learned in the laboratory are reproduced safely in the patient.

Despite the EHS taking a pragmatic approach to robotic hernia training, the feasibility of adhering to the pathway and outcomes of patients during the RAWS training journey have not been reported from the UK. Accordingly, we report the outcomes of consecutive robotic hernia procedures after established laparoscopic surgeons from a single centre that adhered to the EHS recommendations and discuss both its practicality and feasibility within the UK where RAWS is in the early phase of adoption.

## Materials and Methods

### Study Design

This prospective, single-centre, observational cohort study analysed outcomes of RAS abdominal wall hernia surgery over a consecutive series of operations at the Royal Derby Hospital, Derby, UK. The study was approved by the local hospitals clinical audit department (UHDBS391) and took place between November 2021 and September 2024.

### EHS Robotic Abdominal Wall Surgery Pathway

The pathway is designed in phases and can be used to implement a series of techniques for RAWS. These are performed in a stepwise manner with increasing complexity.

### Training for Robotic Surgery

The daVinci X robotic platform (Intuitive, Sunnyvale, CA, US) was used for all procedures. All operations were performed by two surgeons (IB and AA) whose RAWS training began at the start of the study. Both surgeons had between 5 and 10 years of experience in laparoscopic and open hernia surgery prior to embarking on their RAWS journey, including laparoscopic TAPP inguinal hernia, IPOM plus for ventral hernia repair and open TAR. The surgeons had no previous experience in performing laparoscopic TARUP/eTEP or laparoscopic TAR. Both surgeons had the absolute minimum required robotic access (two theatre sessions per surgeon per month) and had clear aims that were set to gain skills in performing RAWS.

**FIGURE 1:**
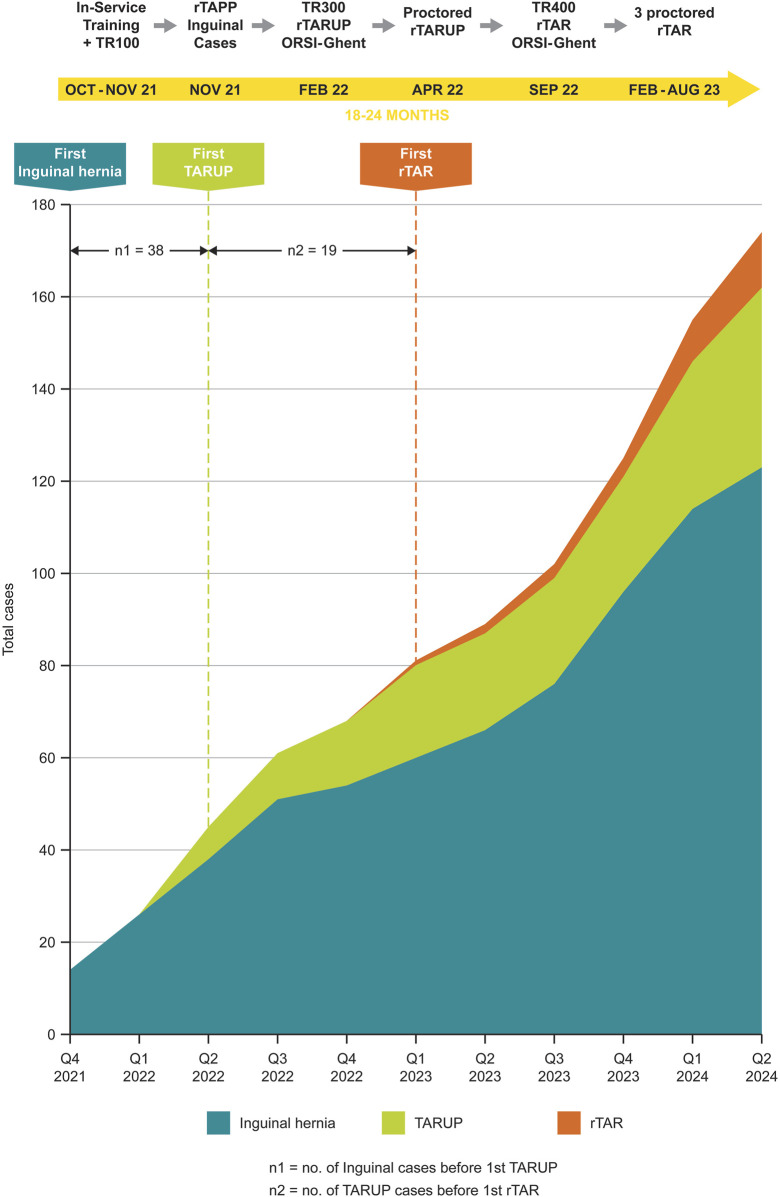
Robotic hernia surgeries performed at the Royal Derby Hospital, UK, showing the progression in robotic surgical complexity over time.

In 2021, prior to their first robotic case, both surgeons (IB and AA) completed preclinical phases I and II training (Figure 1). This involved certification for the daVinci X system, which included completion of the Intuitive online learning modules, >30 h of training on the daVinci Sim Now simulator, case observation at the robotic hernia epicenter, da-Vinci In-Service, da-Vinci Skills Drills with Kit (Intuitive Surgical Ltd, Oxford, UK), and completion of a TR100/200 course in October/November 2021. The TR100/200 course involved performance and assessment of simulator tasks, placement of ports, docking, loading of instruments and safe use of instruments on a live animal model (The Griffin Institute, London, UK), and performing the procedural steps for an rTAPP inguinal hernia repair. Both surgeons (IB and AA) attended webinars and conferences, reviewed the Intuitive procedure guides and frequently reviewed technical procedural videos of RAWS.

Upon successful completion of the initial proctored cases (n = 4) of uncomplicated rTAPP inguinal hernias, each surgeon completed 19 inguinal rTAPP cases independently. The surgeons then attended an advanced TR300 course in ORSI, Belgium, in February 2022 (Figure 1), performing rTAPP inguinal and rTARUP procedures on Kindheart models. This was followed by performing rTARUP cases in April 2022, initially proctored (n = 4) and then 10 cases each independently. Finally, a TR400 course was completed in ORSI, Belgium, in September 2022, followed by attendance at the first EHS robotic ventral hernia course (also in ORSI, Belgium) in November 2022, where rTAR was performed on a live animal model. Subsequently, two in-person, proctored rTAR cases were performed, then a tele-mentored rTAR procedure. All surgical techniques used are as described in the Supplementary Appendix.

The surgeons (IB and AA) had proctor alignment calls with all visiting proctors to discuss the cases, review their scans and confirm that all preferable equipment, sutures and meshes were available.

### Inclusion/Exclusion Criteria

All patients were fit to undergo a general anaesthetic. For TARUP patients a Carbonell equation of ≥2 was determined by CT imaging. For TAR patients, those with a loss of domain >20% (calculated using the Tanaka method) or previous fistula and current stoma were excluded.

### Outcomes Assessed

For analysis, the demographics and preoperative characteristics of all consecutive patients were documented at baseline and grouped according to surgical procedure. Operative data recorded included operative time, imbrication of transversalis fascia (direct rTAPP inguinal hernia repair), size and location of the hernia defect (ventral hernia), conversion to open surgery, reasons for conversion, and length of hospital stay. Postoperative outcomes included general complications (urinary retention, hospital acquired pneumonia, deep vein thrombosis, etc.), hernia-related complications (seroma, haematoma and mesh infection), use of analgesics (opiates), occurrence of day-case surgeries, readmission, and recurrence rates. All complications were graded using Clavien-Dindo classification. Results are reported as number (percentage) or median (interquartile range). To evaluate the learning curve for rTAPP inguinal and rTARUP hernia repairs, operative times were plotted against case numbers, with a 5-case moving average applied to identify the proficiency plateau, defined as stabilisation within a 10% range of the median operative time for at least 5 consecutive cases.

## Results

In total, 144 consecutive patients were operated using RAS (Table 1). Figure 1 illustrates our progress in robotic surgical complexity over time after following the EHS recommendations.

**TABLE 1:** Demographic and preoperative characteristics of consecutive patients undergoing hernia repair using robotic surgery.

Parameters	Hernia repair technique		
	Inguinal rTAPP	rTARUP/eTEP	rTAR
Demographics	(n = 97)	(n = 42)	(n = 13)
Age (years), median (IQR)	61 (48–70)	56 (47–64)	55 (44–65)
Sex, n Male Female	91 6	28 14	7 6
Ethnicity, n White Black Asian British	86 0 11	39 0 3	11 1 1
BMI (kg/m^2^), median (IQR)	26 (24–28)	32 (29–36)	33 (30–35)
ASA classification, median (IQR)	2 (2–2)	2 (2–2)	2 (2–3)
CCI, median (IQR)	2 (1–3)	2 (1–3)	2 (1–3)
Preoperative characteristics			
Preoperative imaging, n Yes No	13 84	22 20	13 0
Indication for repair, n Symptomatic Incarcerated	96 1	42 0	13 0
Type of hernia, n Primary Recurrent Incisional	79 16 —	31 9 2	0 4 9
Type of primary repair, n Open Suture Mesh	16 0 16	9 7 2	0 4 0
Side of groin hernia, n Right Left Bilateral	42 32 23	—	—
EHS classification, n M1M2, W2 M2, W1 M2M3, W2 M2M3, W3 M2-M4, W2 M2-M4, W2, L3, W2 M3, W1 M3, W2 M3, W2, L3, W2 M3-M5, W2 M3-M5, W3 M4M5, W2 M5, W1 L3, W1		1 1 - - - - 38 1 - - - - 1 -	- - 2 1 2 1 - 2 1 1 1 1 0 1
Maximum defect size (cm), median (IQR) Width Height	—	3.2 (2.3–4.1) —	7.0 (5.12–7.54) 11.0 (5.75–15.1)
Loss of domain, median (IQR) Tanaka method	—	—	12.5 (11.2–16.6)

*ASA, American Society of Anesthesiologists; BMI, body mass index; CCI, charlson comorbidity index; EHS, European Hernia Society; IQR, interquartile range; rTAPP, robotic transabdominal preperitoneal; rTARUP, robotic transabdominal retrorectus umbilical prosthetic; eTEP, extended totally extraperitoneal; rTAR, robotic transversus abdominus release.*

### rTAPP Inguinal Hernia

Overall, 97 patients underwent rTAPP inguinal hernia repair, of which 23 were bilateral (Table 1). The median (IQR) age was 61 (48–70) years and median (IQR) BMI 26 [13–17] kg/m^2^. Sixteen cases were recurrent hernias, either following Lichtenstein repair (n = 13) or open repair with preperitoneal mesh placement (n = 3). Four patients had previous open (n = 1) or robotic prostatectomy (n = 3). The mesh was explanted in three cases where a preperitoneal mesh had been placed with previous open repair. The median (IQR) operating time after docking of the final port was 56 (48–72) min for a unilateral inguinal hernia and 101 (82–139) min for a bilateral inguinal hernia (Table 2).

**TABLE 2:** Operative data in consecutive patients undergoing hernia repair using robotic surgery.

Parameters	Hernia repair technique		
	Inguinal rTAPP (n = 120)	rTARUP/eTEP (n = 42)	rTAR (n = 13)
Operative time (min), median (IQR)			
Unilateral	56 (48–72)	167 (147–190)	426 (369–548)
Bilateral	101 (82–139)	—	—
Proficiency plateau case number, n Unilateral Bilateral Type of TAR	20 15 —	18 — —	— —
Unilateral			4
Unilateral inguinal hernia			1
Bilateral			9
Bilateral inguinal hernia			1
Imbrication of transversalis fascia, n Yes No	13 107	—	—
Conversion to open, n Yes No	1 98	1 41	0 13
Reason for conversion, n Large sliding hernia Bigeminy intraoperatively	1 0	0 1	—

IQR, interquartile range; rTAPP, robotic transabdominal preperitoneal; rTARUP, robotic transabdominal retrorectus umbilical prosthetic; eTEP, extended totally extraperitoneal; rTAR, robotic transversus abdominus release.

**FIGURE 2:**
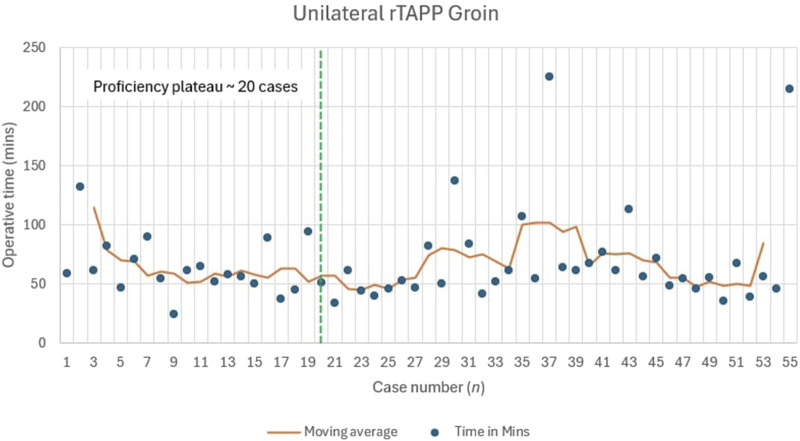
Learning Curve for Unilateral rTAPP Inguinal Hernia Repair.

The moving average for unilateral inguinal hernia showed initial variability, with times ranging from 47 to 137 min in the first 10 cases. Stabilisation occurred after approximately 20 cases, where the moving average fluctuated between 45 and 60 min (within 10% of the median) for cases 21–55. This indicates that surgeons reached a proficiency plateau after 20 cases (Figure 2).

**FIGURE 3:**
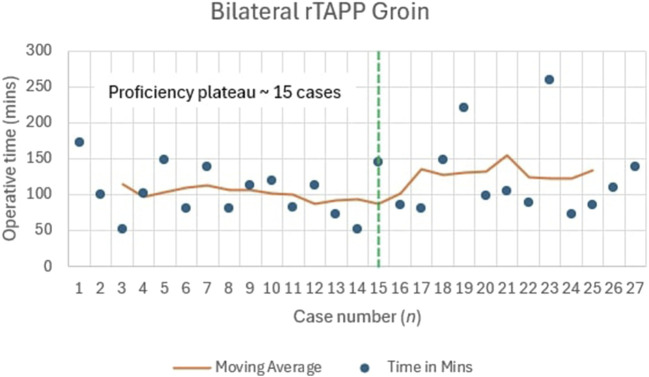
Learning Curve for Bilateral rTAPP Inguinal Hernia Repair.

The moving average for bilateral inguinal hernia indicated high variability in the first 10 cases (72–173 min), stabilising after approximately 15 cases, where times ranged between 80 and 110 min for cases 16–27. This indicates a plateau in operative time after 15 cases (Figure 3).

One patient had an emergent incarcerated inguinal hernia repair. Thirteen patients underwent imbrication of the transversalis fascia when surgeons encountered a large direct hernia defect (Table 2). A patient had to be converted to open surgery when a large sliding inguinal hernia containing sigmoid colon was encountered.

Four (3.3%) patients experienced complications (Table 3). Eighty (82%) patients had day-case discharge, and the remainder were discharged within 24 h. Four (4.1%) patients were re-admitted; two (2.1%) had recurrence at the 6-month follow-up, requiring further repair using the open Lichtenstein technique.

**TABLE 3:** Operative data in consecutive patients undergoing hernia repair using robotic surgery.

Parameters	Hernia repair technique		
	Inguinal rTAPP (n = 97)	rTARUP/eTEP (n = 42)	rTAR (n = 13)
Clavien-Dindo, n 0 I II	93 2 2	38 0 4	11 1 1
Complications, n Vasovagal episode UTI Port site pain Confusion Seroma Mesh infection	1 1 1 0 1 0	0 0 0 0 3 1	0 0 0 1 1 0
Opiate required postoperative, n Yes No	8 89	2 40	13 0
Urinary retention, n Yes TWOC No	4 4 93	NA	NA
Length of stay (days), median (IQR)	0 (0–1)	1 (0–1)	3 (3–4)
Day-case discharge, n Yes No	80 17	18 24	0 13
Readmission, n Yes No	4 93	2 40	0 13
Reason for readmission, n Constipation PPI-induced nephritis Surgery for recurrence Drain removal Infected seroma	1 1 2 0 0	0 0 0 1 1	—
Recurrence, n Yes No	2 95	0 42	0 13
Duration of follow-up (days), median (IQR)	—	6 (4–12)	12 (5–12)

IQR, interquartile range; PPI, proton-pump inhibitors; TWOC, trial without catheter; UTI, urinary tract infection; rTAPP, robotic transabdominal preperitoneal; rTARUP, robotic transabdominal retrorectus umbilical prosthetic; eTEP, extended totally extraperitoneal; rTAR, robotic transversus abdominus release.

### rTARUP

Forty-two patients underwent rTARUP (n = 41) or eTEP (n = 1) (Table 1). The median (IQR) age was 56 (47–64) years, and the median (IQR) BMI was 32 (29–36) kg/m^2^. Thirty-one cases were primary, nine were recurrent, and two were incisional hernias. Seven recurrent hernias had an open suture repair, and two had an open repair with suture and placement of a mesh in the preperitoneal space. The median (IQR) defect size was 3.2 (2.3–4.1) cm wide.

**FIGURE 4:**
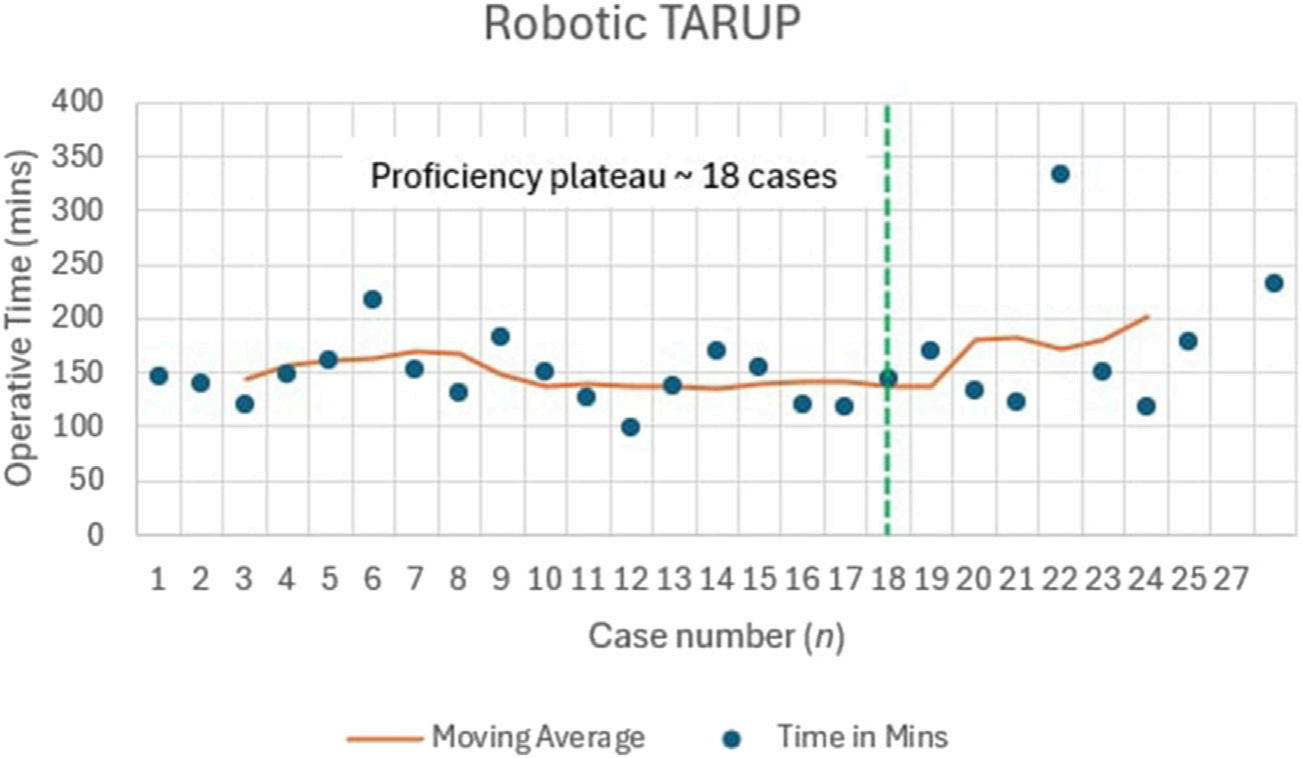
Learning curve for robotic TARUP repair.

The median (IQR) operative time was 167 (147–190) min (Table 2). The moving average showed significant variability in the first 15 cases (118–217 min), stabilising after approximately 18 cases, where times ranged between 130 and 160 min for cases 19–27. This indicates a plateau in operative time after 18 cases (Figure 4).

One patient required conversion to open surgery due to intraoperative cardiac instability. Eighteen patients had a day-case discharge, twenty-two were discharged within 24 h of admission, and the remaining two were discharged at 48 h.

Two (4.8%) patients needed postoperative opiates for pain control (Table 3). One patient presented with an infected seroma that was treated with intravenous antibiotics. Subsequent imaging at the 2-year follow-up confirmed no clinical sign of mesh infection or recurrence. Two patients had a seroma that resolved spontaneously without intervention. No patients had signs of recurrence at the 6-month follow-up.

### rTAR

Thirteen patients underwent TAR (Table 1). The median (IQR) age was 55 (44–65) years, with a median (IQR) BMI of 33 (30–35) kg/m^2^. All patients had incisional hernias. The median (IQR) defect size was 7.0 (5.12–7.54) cm wide and 11.0 (5.75–15.10) cm long. The median (IQR) loss of domain using the Tanaka method was 12.5% (11.2%–16.6%).

Three hernias were lateral L3 hernias (two post-appendicectomy and one post-reversal of an ileostomy). The patient with an L3 hernia post-reversal of ileostomy had a midline component to the hernia (M2-4, W2). The remainder of the cases were midline incisional hernias, varying in location from M2-5 with a width W2-W3.

The median (IQR) operating time was 426 (369–548) min (Table 2). All patients required postoperative opiate analgesia (Table 3). One patient developed postoperative opiate-related confusion, while another developed a postoperative seroma. No patients developed complications that required further intervention or a re-visit to theatre. The median (IQR) length of stay was 3 (3-4) days. No patients had any clinical signs of recurrence at 6 months (seven patients) or 12 months (four patients).

## Discussion

In our series of consecutive patients undergoing robotic inguinal and ventral hernia (rTAPP/rTARUP/rTAR) repair, implementation of the EHS training pathway for RAWS resulted in a low complication rate and satisfactory clinical outcomes. Given that both surgeons were experienced laparoscopic hernia surgeons, initiating the pathway with a low-complexity procedure (grade 1/2 rTAPP inguinal hernia repair) and anatomical familiarity allowed them to concentrate on the nuances of using the robotic platform (i.e., ideal port position, safe application of tension and manipulation of tissue, handling of ProGrip™ mesh, and suturing of the peritoneum with a barbed suture). We believe that use of this low-complexity procedure is ideal for the initiation of RAWS, as cholecystectomy was for laparoscopy especially if surgeons following the robotic training pathway already have previous experience in performing laparoscopic TAPP inguinal hernia repair [18].

The necessity of initially performing a high volume of uncomplicated cases (grade 1/2 rTAPP inguinal hernia repair) enables surgeons to efficiently reach the learning curve, but also to maintain proficiency and skills in using the robotic platform. The stable high-definition, three-dimensional view, combined with improved ergonomics from the wristed instruments, permits surgeons to confidently perform more complex manoeuvres. This allows the safe execution of rTAPP inguinal hernia repair in more complex cases (inguinoscrotal, recurrent with or without the need for mesh excision, and post-prostatectomy inguinal hernia) and enables minimally invasive repair to be offered to a wider cohort of patients [19]. Our recurrence rate was acceptable with only two (1.6%) reported, both in large direct hernias, where non-gripping mesh (ULTRAPRO™) without fixation was placed or imbrication of the transversalis fascia was not performed [20].

The EASTER study reported no difference in the proportion of patients’ post-prostatectomy, with recurrence, or grade 3 inguinal hernias undergoing laparoscopic or robotic hernia repair where surgeries were performed by a specialist hernia surgeon with a high-volume practice [19]. In our practice after reaching the learning curve, surgeons were able to perform more complex inguinal hernia cases using RAS [post-prostatectomy, n = 4/120 (3.3%); recurrence with excision of preperitoneal mesh, n = 3/120 (2.5%); grade 3 inguinal hernia, n = 28/120 (23.3%)], which previously would rarely have been attempted using the laparoscopic approach.

In the UK, there are two key issues related to hernia surgery: first, the average surgeon only performs one elective operating list per week (compared with three lists per week in Europe and 2.5 lists per week in the United States), leading to an overall lower volume of cases; secondly, the volume of hernia cases in the UK are too large for such procedures to be performed solely by a pure specialist hernia surgeon [21, 22]. To add to the quandary, an increase in operating volumes is associated with competence, improved clinical outcomes and efficiency [23–25]. The increased volumes observed from a dedicated hernia specialist in Europe may have been the reason for not observing a difference in outcomes when performing more complicated inguinal hernia surgery between the laparoscopic or robotic approach [19]. It is our opinion that the robotic platform, when used in the context of a dedicated team with an interest in developing a practice in RAWS in conjunction with adherence to the EHS abdominal wall surgery pathway, may help to bridge the training/experience gap and reduce the learning curve for general surgeons that have limited robotic access in performing more complex cases with the MIS approach. Such an effect may lead to standardisation of clinical outcomes.

The rTARUP hernia repairs within our centre were performed after attending an TR300 course in ORSI, Belgium, in February 2022, followed by four proctored cases (four in April 2022) (Figure 1). This was followed by completion of 42 rTARUPs/eTEP with a median defect width of 3.2 (2.3–4.1) cm. Muysoms et al. reported outcomes and operative times for 41 consecutive patients who underwent rTARUP over a 5-month period [2]. The median defect size was 2.15 cm and only 1/41 (2.4%) patients required analgesics, compared to 2/42 (4.8%) patients needing opiates in our cohort. This might be explained by the greater tension needed to close the larger defect in our cohort. A case of infected skin presented in the Muysoms et al. series was treated with drainage and antibiotics, similar to spontaneous drainage and antibiotics in our series, without the need for mesh removal in either case [2]. We reported this as an infected seroma, most likely due to the overzealous use of diathermy between the sac and umbilical skin, causing necrosis leading to superficial skin infection and eventual spread into the seroma. At follow-up, there were no clinical signs of discharge from a chronic sinus and a computed tomography scan confirmed no evidence of recurrence or deep-seated residual infection. The operative times for rTARUP were shorter in the study by Muysoms et al., with a mean operating time of 73 min compared with a median of 167 min in our cohort [2]. The authors reported that the new lateral muscular dissection and stepwise increase in complexity, beginning with rTAPP inguinal hernia repair, contributed to the short time of surgery rather than increased proficiency with using the robotic platform. The time taken to perform a TARUP plateaued showing less variability after the 18th patient in our case series but did not confirm as significant of a time reduction published in other series [2]. This may have been due to both a larger median defect size (3.2 cm vs. 2.15 cm) and lower accessibility to robotic theatres leading to a lower volume of robotic cases performed in the UK [2, 20].

The first proctored rTAR at our centre was performed in February 2023 after completion of a TR400 course in September 2022 (Figure 1). A further two rTARs were performed with a proctor, while the third was performed using teleproctoring technology (Intuitive Hub). Following these cases, the next 10 rTARs were performed independently. Outcomes from our early series, including length of stay (median 4 days vs. 3.4 days^9^) have been encouraging and equivalent to those reported in the literature [9, 26]. Adhesiolysis contributed to the longer operative time duration observed in our series (median time 390 min vs. mean time 242 min^9^), with the remainder remaining constant for dissection of the posterior rectus, posterior component separation, closure of the anterior/posterior layers and placement of mesh. Furthermore, the mean time reported by Dewulf et al. was over the course of performing 90 cases, which may have been due to improvement in both efficiency and competent use of the robotic platform [9]. Bittner et al. reported a similar average operating time to this study (365 min) for their first 26 rTAR cases [27]. We did not encounter any intraoperative adverse events such as bowel injury or bleeding with or without major vessel injury or pleural injury, which may have been the result of performing fewer complex cases in our early series. None of our cases had previous enteric fistula, current stoma, or a loss of domain >20%. We believe that dual surgeon operating and appropriate case selection, in conjunction with learning from expert experience, reduced the risk of encountering such adverse events. Recurrence after rTAR was not noted in our case series, although this could be explained by a shorter median follow up of 12 months vs. 19 months in Dewulf et al. study [9].

Our analysis indicated proficiency plateaus at approximately 20 cases for unilateral rTAPP (median 55 min), 15 cases for bilateral rTAPP (median 101 min), and 18 cases for rTARUP (median 147.5 min). In contrast, Ephraim et al. reported a large series of robotic inguinal hernia repairs with a mean operative time of 51.8 min but did not specify a case threshold for proficiency, suggesting variability in learning curves across settings [28]. Kudsi et al. analysed robotic ventral hernia repairs, including rTAR, noting stable outcomes after 50–100 cases, a higher threshold than our rTAR experience (not fully quantified here due to limited cases, n = 13), which could possibly be due to greater case complexity in their cohort [13]. Korneffel et al. found a learning curve plateau for robotic eTEP hernia repair at 12 cases (operative time reducing from 262 to 192 min), slightly lower than our rTARUP plateau, potentially reflecting differences in procedural complexity or prior surgeon experience [14]. These comparisons highlight that our UK findings align with international trends, though limited robotic access in the NHS may extend learning curves compared to high-volume centres, underscoring the need for structured training pathways to achieve proficiency efficiently.

There were no known proctors for rTAR with GMC registration in the UK at the time that surgeons (IB and AA) were ready to perform their first case. An experienced Swiss proctor was contacted and agreed to apply for temporary GMC registration for visiting eminent specialists; application involved the proctor attaining a certificate of introduction from the Royal College of Surgeons 2 months prior to the visit [15]. The temporary registration was achieved on 8 February 2023, allowing a 6-month period of visitation to the surgeons’ hospital. This allowed the proctor to observe one rTAR in person in February 2023, and another telementored procedure in May 2023. A final case was performed with a proctor who was visiting UK for a fellowship in August 2023. All subsequent cases were performed independently. In October 2023, one of the surgeons (AA) became the first UK-based Intuitive hernia proctor and has proctored one other centre in the UK for both rTARUP and rTAR. This achievement will contribute to the safe propagation of the EHS abdominal wall surgery pathway in the UK.

An important aspect of training that the EHS abdominal wall surgery pathway does not address is maintenance of proficiency. There is reported evidence supporting the notion that an increase in volume is associated with proficiency and better patient outcomes. The Royal College of Surgeons has addressed the minimum number of cases that should be performed in their guidance, “Robotic Assisted Surgery: A Guidance to the Future” [16]. The authors suggest a minimum of 20–25 cases per annum over a 2-year period, in addition to completion of five core simulator skill exercises achieving more than 90% at the end of the 2-year period. Performing 50 cases over a 2-year period would make the surgeon exempt from the need to complete the simulator skill exercises. A minimum number is set to maintain a standard to avoid patient harm. However, a surgeon should aim to attain greater numbers to achieve excellence. A case mix of low-complexity, high-volume procedures (inguinal hernia: post-prostatectomy, recurrence post-open repair, inguinoscrotal), moderate-complexity, moderate-volume procedures (ventral hernia 2–5 cm, rTARUP), and high-complexity, low-volume procedures (ventral hernia >5 cm, rTAR) may permit sufficient case volume to maintain proficiency with a view to achieving excellence. This must be offset against a landscape in the NHS where a surgeon has access to one theatre list per week, waiting lists are increasing [17], and evidence is accumulating that demonstrates superior outcomes with robotic hernia surgery. Unless surgeons show dedication, focussing on moving towards greater theatre access (as observed in the USA and rest of Europe), proficiency and quality will be lacking, forcing non-clinical and robotic-naive clinical managers to assume the robot as an obstacle, rather than a force that will help them achieve their waiting list targets. Increasing theatre access will allow an increase in the number of robotic cases, as well as improved proficiency and better efficiency; an attribute that the NHS is pursuing to manage the long waiting lists. Reducing the waiting list in the UK is certainly multifactorial and complex, but if utilised appropriately, a robotic system can stimulate interest and vigour into a workforce suffering from burnout in the post-Covid era [29, 30].

To ensure the feasibility of adopting RAWS within the NHS, addressing financial sustainability, infrastructure requirements, and workforce implications is critical. Financially, RAWS involves substantial upfront costs for robotic systems (approximately £1.5–2 million per unit) and ongoing expenses for maintenance and consumables, which must be balanced against potential increased efficiencies in operative times, reductions in hospital stay and complications, as evidenced by studies reporting decreased operating time with experience and case volume, decreased length of stay and lower wound complication rate with robotic hernia repair [8, 31–33]. Infrastructure demands include dedicated operating theatres equipped with robotic platforms and space for training facilities, posing challenges in resource-constrained NHS hospitals. Workforce implications necessitate a skilled cadre of surgeons, requiring extensive training often funded by industry which raises concerns about cost sustainability and potential bias. Industry-funded training, while facilitating access to advanced courses like TR100–400, may strain NHS budgets if long-term funding shifts to public resources, necessitating transparent cost-sharing models to maintain accessibility without compromising ethical standards.

Whilst other UK centres have indeed developed effective robotic AWR programmes independently, these have generally been institution-specific initiatives. For example, Ward et al. reported that a UK NHS unit safely introduced robotic Rives–Stoppa repairs (28 cases) with no increase in operative time or mortality, shorter hospital stays, and significant cost savings compared to open repair [34]. Such results show that skilled teams can attain good outcomes via local routes. It is important to note, however, that Ward et al.’s unit had an established high-volume open AWR practice prior to robotic adoption, which likely contributed to their ability to implement robotic techniques safely and effectively. By contrast, the EHS pathway prescribes a structured, phased curriculum – preclinical simulation and company-led console training, followed by mentored initial cases and gradual escalation of case complexity – explicitly to ensure competency and patient safety during the learning curve [7]. This standardised framework (analogous to the ACS/EAU robotic curricula) incorporates objective milestones, proctoring, and even an EHS registry for outcome monitoring, which together promote consistency across centres. In resource-limited settings or units with varied surgeon experience, such a replicable training model helps allocate scarce robotic time efficiently and guards against errors early on. In summary, while non-EHS approaches (like Ward et al.’s) demonstrate that high-volume robotic AWR is feasible without the EHS training pathway, we believe this pathway offers a safe, evidence-based blueprint for wider adoption of robotic hernia surgery in the UK, independent of the centres prior experience.

Our study reports the first outcomes of consecutive robotic hernia cases in the UK after adhering to the EHS robotic abdominal wall surgery pathway. The stepwise increase in complexity from rTAPP, rTARUP to rTAR (first case), with proctor involvement at each stage, took place over 18 months. The completion of the pre-clinical phases (1, visit an “epicentre,” advanced cases, and discussion of clinical and economic benefits; 2, secured access, online modules, simulation tasks over 20 h, review of procedure cards, and attendance of TR100-400 courses) and clinical phases (3, initial case performed with a proctor; 4, continuing development) were essential for performing safe procedures with equivalent to reported outcomes.

There are some limitations to this study. Firstly, it was conducted at a single centre with a relatively small sample size, particularly for rTAR procedures (n = 13), which may limit the generalisability of the findings to other UK or international settings with different surgical volumes or resources. Secondly, the follow-up duration varied across procedures, with rTAPP and rTARUP patients followed for 6 months and rTAR patients for up to 12 months, potentially underestimating long-term recurrence rates or complications. Lastly, the study relied on observational data without a control group, such as patients undergoing laparoscopic or open hernia repair, precluding direct comparisons of outcomes between techniques.

## Conclusion

Adherence to the EHS robotic abdominal wall surgery pathway is feasible in the UK and represents a robust mechanism for surgeons interested in complex abdominal wall surgery to achieve safe and satisfactory outcomes.

## Data Availability

The raw data supporting the conclusions of this article will be made available by the authors, without undue reservation.
