# Supplementary material for: Outcomes During the Learning Curve and Feasibility of Implementing the European Hernia Society Recommendation Guidelines for Robotic Abdominal Wall Surgery Within a UK Centre
Source: J Abdom Wall Surg. 2025 Aug 6;4:15008. doi: 10.3389/jaws.2025.15008 (PMC12364726; doi:10.3389/jaws.2025.15008)
Supplement: Supplementary file 1 [file Supplementaryfile1.docx]

**Outcomes during the learning curve and feasibility of implementing European Hernia Society recommendation guidelines for robotic abdominal wall surgery within a UK centre**

**Supplementary Materials - Index**

| **Supplementary Appendix** |  |
| --- | --- |
| Surgical Methods | *pag. 2* |
| References | *pag. 4* |

**Supplementary Appendix: Surgical Methods**

***Robotic transabdominal preperitoneal (rTAPP) inguinal hernia repair***

A closed pneumoperitoneum was created using a Veress needle (Locamed, UK) inserted at Palmers point. During the first 20 cases of rTAPP inguinal hernia, an assistant 12mm Airseal (Conmed, New York, USA) optical port was placed in the left upper quadrant, 10cm above the umbilicus in the mid clavicular line. The assistant Airseal (Conmed, New York, USA) port was used to pass the V-Loc™ sutures (Medtronic, Dublin, Ireland), ULTRAPRO^TM^ 15x10cm (first 20 cases) or ProGrip™ 15x10cm mesh (Medtronic, Dublin, Ireland) (after case 20) and 15x2.5cm (16 Ply) laparoscopic swabs (Unisurge, Newmarket, UK). This was followed by insertion of three 8mm robotic ports, 8cm distant from each other in a horizontal line, just above the level of the umbilicus (Intuitive, Sunnyvale, California, USA). Beyond 20 cases, the surgeons did not use an Airseal optical (Conmed, New York USA) port and the mesh, suture and laparoscopic swabs were passed through an 8mm robotic port. It is now routine practise to use three 8mm robotic ports once a closed pneumoperitoneum has been created using the Veress needle. An 8mm optical robotic port (Intuitive, Sunnyvale, Ca, USA) was inserted under vision using a 5mm laparoscope (Karl Storz, Germany), immediately above the umbilicus; two 8mm robotic ports (Intuitive, Sunnycale, Ca, USA) were then placed 8cm apart on either side. The left 8mm robotic port (Intuitive, Sunnycale, Ca, USA) had an Airseal cap (Conmed, New York, USA) and was connected to the Airseal insufflator (Conmed, New York, USA). Before docking, the patient was placed in a 20-degree Trendelenburg position. Robotic arms 1 (robotic fenestrated bipolar forceps) & 3 (robotic scissors and robotic needle driver) were used as working ports, and robotic arm 2 for a 30-degree endoscope. A preperitoneal plane was dissected, the hernia sac was reduced, and a critical view of the myopectineal orifice was achieved.^1^ Large direct hernia defects were closed with a 3/0 absorbable V-Loc™ (Medtronic, Dublin, Ireland) by imbricating the transversalis fascia, carefully taking small bites to avoid injuring the cord structures running anteriorly. A 15x10cm ProGrip™ mesh was placed in the dissected pocket. The peritoneum was closed with a 3/0 absorbable V-Loc™ suture (Medtronic, Dublin, Ireland).

***Robotic transabdominal retromuscular umbilical prosthetic (rTARUP) hernia repair***

All patients had a preoperative CT abdomen. The Carbonell equation was used to calculate the ratio between the sum of the width of the recti and the width of the hernia defect. Only hernias with a Carbonell ratio^[[1]](#footnote-1)^ ≥2 and defects ≤5cm were considered for rTARUP. Furthermore, the hernia defects were situated in the M2-M4 region of the midline^2^ and were either primary or incisional hernias. A closed pneumoperitoneum was created using a Veress needle (Locamed, UK) inserted at Palmers point and a pressure of 12mmHg was established. A 15x15cm square was drawn, centred on the hernia defect. When approaching cases with multiple hernia defects, a 20x15cm rectangle was marked on the abdomen. The marking was important to guide the direction and extent of the initial incision of the ipsilateral posterior rectus sheath (PRS), the extent of the dissection of the crossover of the midline, and the extent of dissection of the contralateral PRS. This pocket was necessary to allow placement of a 15x15cm or 20x15cm ProGrip™ mesh (Medtronic, Dublin, Ireland), depending on the extent of dissection. Three points were marked, at a minimum of 7cm from the left edge of the marking and at least 8cm apart to guide insertion of the 8mm robotic ports (Intuitive, Sunnyvale, Ca, USA). The 8mm robotic port was used as an optical trocar and was placed in the marking in the left upper quadrant using a 5mm laparoscope (Karl Storz, Germany). The two remaining ports were placed in the markings made on the left abdominal flank. Robotic arms 1 (robotic fenestrated bipolar forceps) and 3 (robotic scissors and robotic needle driver) were used as working ports, while arm 2 was used for the Robotic 30-degree endoscope. A hypodermic needle was placed along several points on the left edge of the markings to guide the direction and extent of initial dissection of the ipsilateral PRS. The ipsilateral PRS was dissected to the decussating fibres where the posterior and anterior rectus sheath (ARS) meet to form the linea alba. An incision was made 5mm away the decussating fibres of the ipsilateral PRS to expose the preperitoneal fat beneath and linea alba above. The preperitoneal fat in the hernia defect was reduced when possible and, in recurrent or incisional hernias, the adherent sac was divided. Dissection was pursued until the right rectus muscle was observed through the contralateral PRS. The contralateral PRS was divided and dissected off the right rectus muscle. The extent of dissection was guided by puncture of the hypodermic needle through markings on the abdomen but was limited to avoid injury to the right sided neurovascular bundles. The abdominal pressure was lowered to between 6-8mmHg and the hernia defect was closed with a 0 absorbable V-Loc™ suture (Medtronic, Dublin, Ireland). In select cases with hernia defects ≥3cm and a wide divarication, closure also included 4cm of linea alba on either side to improve the robustness of the repair. A ProGrip™ (Medtronic, Dublin, Ireland) mesh was placed in the pocket against the recti muscles. The access incision on the ipsilateral PRS was closed with a 3/0 absorbable V-Loc™ suture (Medtronic, Dublin, Ireland). Peritoneal defects were closed with a 3/0 absorbable V-Loc™ suture (Medtronic, Dublin, Ireland) or 4/0 Vicryl (Ethicon, Johnson & Johnson, UK). In a single case where the peritoneum could not be closed, the defect was bridged with a Bio-A® biosynthetic mesh (Gore®, Delaware, USA) and sutured in an interrupted onlay fashion to the peritoneum with a 4/0 Vicryl (Ethicon, Johnson & Johnson, UK).

In patients with a smaller abdomen and a midline hernia either in the M1/M2 or M4/M5 position, inferior or superior port placement/docking was used, respectively. Again, a 15x15cm square was marked, centred on the hernia defect. Three robotic ports were placed in a straight line at least 7cm from the nearest edge of the drawn square and 8cm apart. The instrument used was unchanged from standard TARUP. With this approach, the left and right PRS were divided and the muscles on either side were exposed (muscle anterior and PRS inferior). The medial aspect of the PRS was divided 5mm away from the linea alba to expose in the midline the preperitoneal fat below and the linea alba above. Lateral dissection of the PRS was limited to avoid injuring the neurovascular bundle and semilunar line. Dissection was extended towards the hernia defect, sequentially dividing the medial PRS on either side. The hernia sac was reduced or divided and the extent of dissection (7.5-10cm) beyond the hernia defect was confirmed using the hypodermic needle to puncture the site of the distal abdominal markings. The hernia defect was closed with a 0 absorbable V-Loc™ suture (Medtronic, Dublin, Ireland). A ProGrip™ (Medtronic, Dublin, Ireland) was placed in the dissected space and the peritoneal openings were closed with a 3/0 absorbable V-Loc™ suture (Medtronic, Dublin, Ireland) or 4/0 Vicryl (Ethicon, Johnson & Johnson, UK). The access incision on the ipsilateral PRS was closed with a 3/0 absorbable V-Loc™ suture (Medtronic, Dublin, Ireland).

***Extended totally extraperitoneal (eTEP) TARUP hernia repair***

This approach was used in primary or incisional hernia defects in the M3/M4/M5 position. A 5mm Excel port was used as an optical trocar in the left upper quadrant over the left rectus muscle to enter the space between muscle and PRS. A space was dissected in this plane by both insufflation to 12mmHg and dissection with a Johann or hook diathermy to allow the placement of three 8mm robotic ports (Intuitive, Sunnyvale, Ca, USA) at least 8cm apart. The robotic ports were carefully placed through the lateral aspect of the muscle, medial to the neurovascular bundle. The Davinci X robot (Intuitive, Sunnyvale, Ca, USA) was then docked to the ports. The same procedure was followed as in the description of the TARUP until closure of the hernia defects. The peritoneal defects were then closed with a 4/0 Vicryl (Ethicon, Johnson & Johnson, UK). The ProGrip™ (Medtronic, Dublin, Ireland) mesh was placed and ports were removed after undocking the robot.

***Robotic transversus abdominis release (rTAR ) hernia repair***

This approach was reserved for incisional hernias in the L2/3 region or in the midline M2-M5, with a Carbonell ratio^1^ ≤2 or a defect ≥5cm. Patients with >20% loss of domain were excluded from a robotic operation. A closed pneumoperitoneum was created with a Veress needle (Locamed, UK) inserted at Palmers point with the pressure at 12mmHg. Three robotic 8mm ports (Intuitive, Sunnyvale, Ca, USA) were placed along the left anterior axillary line, 8cm apart. Robotic arms 1 (fenestrated bipolar forceps) and 3 (robotic scissors and robotic needle driver) were used as working ports, while arm 2 was used for the 30-degree robotic endoscope. Adhesions from the abdominal wall were divided and contents (omentum or small bowel) adherent to the hernia sac were dissected and reduced. Dissection of the contralateral PRS was performed adjacent to the hernia defect. The PRS was dissected off the rectus muscle to the neurovascular bundles from the arcuate line to the level of the xiphisternum. Below the arcuate line, the peritoneum was dissected into the Retzius space towards the midline and Bogros space laterally. In female patients, the round ligament was ligated twice, close to the peritoneum, with a 2/0 Vicryl (Ethicon, Johnson & Johnson, UK) suture and divided between the two ligatures. In male patients, the cord structures were protected and parietalised. When encountered with concomitant inguinal hernia, the critical view of the myopectineal orifice was achieved and an additional ProGrip™ (Medtronic, Dublin, Ireland) mesh was placed as previously described. A combination of 'bottom up' and 'top down' TAR was utilised. Dissection was continued lateral to Bogros space, 'caving' into the preperitoneal plane behind the linea semilunaris. The 'bottom up' TAR was performed by dividing the aponeurotic portion of the transversus abdominis between the 'cave' and the PRS, avoiding injury to the neurovascular bundle. The transversus abdominis curved towards the midline superiorly. Superiorly the transversus muscle was divided, and the fascia transversalis was dissected off the muscle to add strength to the peritoneal layer, which is thin and fragile in the upper abdomen. The two approaches were continued to release all the myofascial attachment from the musculo-aponeurotic portion of the transversus abdominis muscle. Dissection in the preperitoneal or pretransversalis plane was continued laterally and superiorly to expose watershed fat and diaphragm. Any defects in the PRS or peritoneum were closed with a 4/0 Vicryl (Ethicon, Johnson & Johnson, UK). Three robotic ports were placed on the contralateral side. The instruments were removed, robotic arms undocked and the table with the patient rotated 180 degrees. The ports were docked to the robotic arms and then instruments placed again. The same procedure was followed on the contralateral side. When approaching the 8mm robotic ports in the undissected side, they were pulled back into the abdominal wall to allow dissection to proceed. The small defects in the peritoneum were closed with a 4/0 Vicryl (Ethicon, Johnson & Johnson, UK). The posterior layer was closed with a 3/0 absorbable V-Loc™ suture (Medtronic, Dublin, Ireland). The pneumoperitoneum pressure was reduced to between 6-8mmHg. The anterior hernia defects were closed with a 0 absorbable V-Loc™ suture (Medtronic, Dublin, Ireland). The space was measured at its maximum dimensions with a sterile ruler (Fannin, Derbyshire, UK). A Versatex™ (Medtronic, Dublin, Ireland) 45x30cm mesh cut to size was placed in the dissected space. No drains were placed in the dissected space.

**References**

1. Daes J, Felix E. Critical View of the Myopectineal Orifice. Ann Surg. 2017;266(1):e1-e2.

2. Muysoms FE, Miserez M, Berrevoet F, Campanelli G, Champault GG, Chelala E, et al. Classification of primary and incisional abdominal wall hernias. Hernia. 2009;13(4):407-14.

1. Alfredo Carbonell’s algorithm, presented at the 9th Annual Abdominal Wall Reconstruction Summit, Montana, USA, 2018. [↑](#footnote-ref-1)
